# Mortality Trends for Neglected Tropical Diseases in the State of Sergipe, Brazil, 1980–2013

**DOI:** 10.1186/s40249-016-0232-8

**Published:** 2017-02-08

**Authors:** Marcos Antônio Costa de Albuquerque, Danielle Menezes Dias, Lucas Teixeira Vieira, Carlos Anselmo Lima, Angela Maria da Silva

**Affiliations:** 1 University Hospital, Federal University of Sergipe, Rua Claudio Batista s/n, B. Santo Antonio, Aracaju, SE 49060-108 Brazil; 2 Post-doc Medicine Unit, Federal University of Sergipe, Rua Claudio Batista s/n, B. Santo Antonio, Aracaju, SE 49060-108 Brazil

**Keywords:** Mortality, Neglected tropical diseases, Trends, Joinpoint

## Abstract

**Background:**

Neglected Tropical Diseases are a set of communicable diseases that affect the population so low socioeconomic status, particularly 1.4 billion people who are living below the poverty level. This study has investigated the magnitude and mortality time trends for these diseases in the state of Sergipe, Northeast Region of Brazil.

**Methods:**

We conducted an ecological study of time series, based on secondary data derived from the Mortality Information System of the Ministry of Health. The mortality rates (crude, age-standardized rates and proportional ratio) were calculated from the deaths due to Neglected Tropical Diseases in the state of Sergipe, from 1980 to 2013. The time trends were obtained using the Joinpoint regression model.

**Results:**

Three hundred six thousand and eight hundred seventy-two deaths were certified in the state and Neglected Tropical Diseases were mentioned as the underlying cause in 1,203 certificates (0.39%). Mean number of deaths was 35.38 per year, and crude and age-standardized mortality rates were, respectively: 2.16 per 100 000 inhabitants (95% *CI*: 1.45–2.87) and 2.87 per 100 000 inhabitants (95% *CI*: 1.93–3.82); the proportional mortality ratio was 0.41% (95% *CI*: 0.27–0.54). In that period, Schistosomiasis caused 654 deaths (54.36%), followed by Chagas disease, with 211 (17.54%), and by Leishmaniases, with 142 (11.80%) deaths. The other diseases totalized 196 deaths (16.30%). There were increasing mortality trends for Neglected Tropical Diseases, Schistosomiasis and Chagas disease in the last 15 years, according to the age-standardized rates, and stability of the mortality trends for Leishmaniases.

**Conclusions:**

The Neglected Tropical Diseases show increasing trends and are a real public health problem in the state of Sergipe, since they are responsible for significant mortality rates. The following diseases call attention for showing greater number of deaths in the period of study: Schistosomiasis, Chagas disease and Leishmaniases. We finally suggest that public managers take appropriate actions to develop new strategies in epidemiological and therapeutic surveillance, and in the follow-up of these patients.

**Electronic supplementary material:**

The online version of this article (doi:10.1186/s40249-016-0232-8) contains supplementary material, which is available to authorized users.

## Multilingual abstracts

Please see Additional file 1 for translations of the abstract into the five official working languages of the United Nations.

## Background

Neglected Tropical Diseases (NTDs) are a set of communicable diseases that affect the population so low socioeconomic status, particularly 1.4 billion people who are living below the poverty level, mainly those located in the Sub-Saharan Africa, Caribbean, Asia, and Latin America [1]. They represent a group of diseases of high global prevalence, namely: Leishmaniases, Schistosomiasis, Onchocerciasis, Lymphatic filariasis, Taeniasis, Cysticercosis, Dracunculiasis, Chagas disease, Human African trypanosomiasis, Leprosy, Dengue, Buruli ulcer, Echinococcosis, endemic treponematoses (Yaws, Pinta, Bejel), Rabies, Trachoma, foodborne trematodiases and some soil-transmitted helminthiases (*Ascaris lumbricoides*, *Trichuris trichiura* and Hookworm infection) [2]. These diseases are so called because the research investments are generally not reverted to development and amplification of access to new drugs, diagnostic tests, vaccines and other technologies for their prevention and control [3].

Globally, Hookworm infection is the most prevalent neglected tropical disease (NTD), and the number of deaths due to Leishmaniasis, Schistosomiasis and human African trypanosomiasis (HAT) is extremely high [4]. The highest incidence rate of these diseases in Latin America and the Caribbean occurs in Brazil, where approximately 40 million people live in extreme poverty. Most of Brazil’s poorest people are infected with one or more NTDs, especially either hookworms or roundworms, or both [5]. NTDs are more prevalent in the Northeast and North Region of the country, where we find the lowest Human Development Indices (HDIs). Most NTDs occur in several Brazilian regions; some occur in outbreaks, such as Onchocerciasis, which is limited to the Yanomami indigenous zone (North Region), and lymphatic Filariasis, strongly concentrated in the metropolitan area of Recife [4].

Due to lack of data on mortality impact caused by NTDs in the State of Sergipe, Northeast Region of Brazil, the present paper provides information concerning these neglected diseases, which may contribute to warn managers about the need to prioritize and readjust actions related to surveillance, support, therapeutic quality, and control of these diseases. Here, we analysed the magnitude and mortality time trends for the Neglected Tropical Diseases in the state of Sergipe, Brazil, emphasizing the underlying causes of deaths within the period of study.

## Methods

### Study area

Sergipe is the smaller state of Brazil, total area is 21 918.493km^2^, and accounts for 0.26% of the national total area. It is located in the Northeast, comprising 75 towns, according to the *Instituto Brasileiro de Geografia e Estatística* (IBGE) [6]. The IBGE estimated a population of 2 219 574 inhabitants for Sergipe in 2014, population density is 94.36 inhabitants/km^2^, and life expectancy at birth is 72.1 years [7]. The general climate in the state is semiarid, consisting of seven to 11 dry months. The average annual pluviometric index fluctuates, and irregular and poorly distributed rains occur throughout the year. Waste water treatment is mostly made through septic tanks and part of the effluents generated in the community is released in the general waste water network and, in some cities, directly to the rain water system, without the duly treatment. Approximately 50% of the population lives within the Poverty Index [8]. At least the following NTDs are recognized endemically in Sergipe: Leishmaniases, Schistosomiasis, Chagas disease, Dengue, Leprosy, Taeniasis, soil-transmitted helminthiases (*Ascaris lumbricoides*, *Trichuris trichiura* and Hookworm infection).

### Study design, population and data sources

We designed an ecological study of time series to assess mortality trends for Neglected Tropical Diseases in the state of Sergipe, from 1980 to 2013. Mortality data, which are of public domain, were obtained directly from the Mortality Information System (SIM) for the state of Sergipe, Brazil, and defined by the underlying cause of deaths. The NTDs used, corresponding ICD-9 (1980–1995) and ICD-10 (1996–2013), were obtained from the WHO list [2] and are summarized in Table 1. In addition to joint analysis, the three main ones were analyzed individually. The information regarding population was derived from the demographic census of 1980, 1991, 2000, 2010 and the inter-census estimates, obtained from IBGE.

**Table 1:** Number of deaths, relative frequency and mortality rates of Neglected Tropical Diseases in the state of Sergipe, Brazil, 1980–2013

Neglected Tropical Disease	ICD-9	ICD-10	Number	% (95% CI)	Crude rates (95% CI)	Age − standardized rates (95% CI)	Proportional ratios (95% CI)
Schistosomiasis	120	B65	654	54.36 (51.5 − 57.21)	1.17 (0.65 − 1.70)	1.65 (0.91 − 2.39)	0.22 (0.12 − .32)
Chagas disease	086.0, 086.1, 086.2	B57	211	17.54 (15.43 − 19.81)	0.38 (0.08 − 0.68)	0.57 (0.12 − 1.02)	0.07 (0.02 − 0.13)
Leishmaniases	085	B55	142	11.80 (10.03 − 13.76)	0.25 (0.01 − 0.50)	0.24 (0.01 − 0.48)	0.05 (0.00 − 0.09)
Dengue	061, 065.4	A90, A91	91	7.56 (6.13 − 9.21)	0.16 (0.00 − 0.36)	0.18 (0.00 − 0.39)	0.03 (0.00 − 0.07)
Leprosy	030	A30	57	4.74 (3.61 − 6.10)	0.10 (0.00 − 0.26)	0.15 (0.00 − 0.37)	0.02 (0.00 − 0.05)
Soil-transmitted helminthiases	126, 127.0, 127.2, 127.3	B76, B77, B78, B79	24	2.00 (1.28 − 2.95)	0.04 (0.00 − 0.14)	0.04 (0.00 − 0.12)	0.01 (0.00 − 0.03)
Rabies	071	A82	12	1.00 (0.52 − 1.74)	0.02 (0.00 − 0.09)	0.02 (0.00 − 0.08)	0.004 (0.00 − 0.017)
Cysticercosis	123.1	B69	5	0.42 (0.14 − 0.97)	0.01 (0.00 − 0.05)	0.01 (0.00 − 0.06)	0.002 (0.00 − 0.01)
Yaws	102	A66	2	0.17 (0.02 − 0.60)	0.004 (0.00 − 0.033)	0.004 (0.00 − 0.035)	0.001 (0.00 − 0.006)
Lymphatic Filariasis	125.0, 125.1, 125.2, 125.4, 125.5, 125.6, 125.9	B74	2	0.17 (0.02 − 0.60)	0.004 (0.00 − 0.033)	0.005 (0.00 − 0.041)	0.001 (0.00 − 0.006)
Buruli ulcer	031.1	A31.1	1	0.08 (0.00 − 0.46)	0.002 (0.00 − 0.022)	0.003 (0.00 − 0.034)	NC
Teniasis	123.0, 123.2, 123.3	B68	1	0.08 (0.00 − 0.46)	0.002 (0.00 − 0.022)	0.002 (0.00 − 0.031)	NC
Food borne trematodíases	121	B66	1	0.08 (0.00 − 0.46)	0.002 (0.00 − 0.022)	NC	NC
Human African trypanosomiasis	086.3, 086.4, 086.5	B56	0	0.00 (0.00 − 0.31)	NC	NC	NC
Trachoma	076	A71	0	0.00 (0.00 − 0.31)	NC	NC	NC
Dracunculiasis	125.7	B72	0	0.00 (0.00 − 0.31)	NC	NC	NC
Echinococcosis	122	B67	0	0.00 (0.00 − 0.31)	NC	NC	NC
Onchocerciasis	125.3	B73	0	0.00 (0.00 − 0.31)	NC	NC	NC
Pinta	103	A67	0	0.00 (0.00 − 0.31)	NC	NC	NC
Bejel	104	A65	0	0.00 (0.00 − 0.31)	NC	NC	NC
Total			1,203	100.00	2.16 (1.45 − 2.87)	2.87 (1.93 − 3.82)	0.41 (0.27 − 0.54)

95% *CI* confidence intervals of 95%, *NC* not calculated

### Statistical analysis

Crude mortality rates (CRs) were calculated to assess the annual risk of NTDs deaths in the state population. To compare studies in different locations and populations globally and nationally, we tried to reduce the effect of age differences between populations by calculating age-standardized mortality rates (ASRs). For the standardization, we used the direct method, Brazilian population of 2010 census as standard. The rates were calculated dividing the number of deaths by mid-period yearly population, expressed per 100 000 inhabitants. For the analysis of the age-specific rates, data were grouped into the following age groups: 0 to 19, 20 to 39, 40 to 59, and 60 plus years of age. We also calculated proportional mortality ratios (PMRs), dividing the number of NTD deaths by the total number of deaths, following multiplying by 100.

Trend analyses were performed using the Joinpoint Regression Program version 4.2.0.2 [9], based on the calculation of the annual percent change (APC) and its respective 95% confidence intervals (95% *CI*). Through the segmented log-linear regression, the Joinpoint method enables the description of a trend and the identification of recent changes, modeling linear segments united by inflexion points [10]. The option of heteroscedastic error used to model the trends was the Poisson model. The statistical significance was obtained using the permutation test of Monte Carlo, which through 9 999 permutations, chose the model with the best number of significant Joinpoints. We have also calculated the average annual percent change (AAPC) for the entire period and for the last 15 years [11]. If the APC or AAPC is positive and significant, there is an increasing trend; on the other hand, if the APC or AAPC is negative and significant, the trend is decreasing. If there is no significance, the trend it is considered stable.

## Results

During the study period, we observed 306 872 deaths in the state; the NTDs were mentioned as the underlying cause in 1 203 certificates (0.39%). Average number of deaths was 35.38 per year; average annual mortality rates (CRs, ASRs) were, respectively: 2.16 per 100 000 (95% *CI*: 1.45–2.87) and 2.87 per 100 000 (95% *CI*: 1.93–3.82); the PMR was 0.41% (95% *CI*: 0.27–0.54). Table 1 shows the main NTDs responsible for mortality, which were: Schistosomiasis, 654 deaths (54.36%), followed by Chagas disease, 211(17.54%) and Leishmaniases, 142 (11.80%). The other diseases totalized 196 deaths (16.30%).

**Fig. 1:**
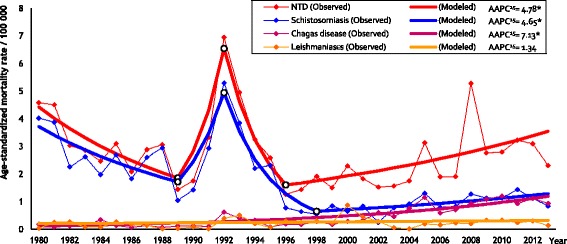
Trends of age-standardized mortality rates by NTDs in the state of Sergipe, Brazil, 1980–2013. * Statistically important. AAPC^15^: Annual percentage change in the last 15 years

**Fig. 2:**
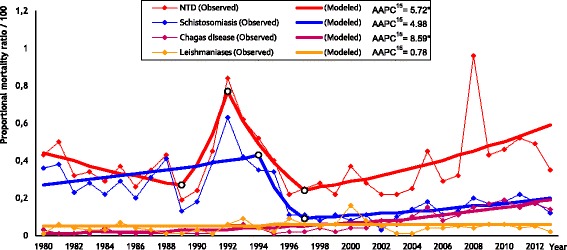
Trends of proportional mortality ratios by NTDs in the state of Sergipe, Brazil, 1980–2013. * Statistically important. AAPC^15^: Annual percentage change in the last 15 years

Mortality time trends for NTDs (ASRs and PMRs), by Joinpoint regression analysis, are showed in Figs. 1 and 2, respectively. We observed increasing temporal trends for NTDs (AAPC^15^: 4.78; 95% *CI*: 1.5–8.1), Schistosomiasis (AAPC^15^: 4.65; 95% *CI*: 0.8–8.7) and Chagas disease (AAPC^15^: 7.13; 95% *CI*: 5.4–8.9) in the last 15 years, according to the age-standardized rates, and stability of the mortality trends for Leishmaniases (AAPC^15^: 1.34; 95% *CI*: -1.3–4.1). Proportional mortality ratios presented significant increases for NTDs (AAPC^15^: 5.72; 95% *CI*: 1.6–10.0) and Chagas disease (AAPC^15^: 8.59; 95% *CI:* 6.8–10.4); and stability for Schistosomiasis (AAPC^15^: 4.98; 95% *CI*: -0.3–10.6) and Leishmaniases (AAPC^15^: 0.78; 95% *CI*: -1.5–3.1) in the last 15 years.

Table 2 summarizes the prevailing epidemiological characteristics of mortality from NTDs, highlighting gender, males (59.98%) and age group, 60 plus (41.36%). Similar results were observed for Schistosomiasis and Chagas disease. For Leishmaniases, age group 0-19 was the most predominant. Crude mortality rates, age-specific rates and proportional ratios have its trends exhibited in Table 3. Increasing trends were observed in the following age groups: 0–19 (AAPC^15^: 3.31; 95% *CI*: 0.3–6.4) and 60 plus (AAPC^15^: 6.46; 95% *CI*: 3.6–9.4). A decreasing trend was observed in age group 20–39 (AAPC^15^: -2.40; 95% *CI:* -4.2;-0.6). The findings in PMRs trends were similar, considering age-specific groups.

**Table 2:** Epidemiological characteristics of mortality from Neglected Tropical Diseases in the state of Sergipe, Brazil, 1980-2013

	Neglected Tropical Diseases			Schistosomiasis			Chagas disease			Leishmaniases		
	N	%	95% CI	N	%	95% CI	N	%	95% CI	N	%	95% CI
Gender^a^												
Male	721	59.98	57.15 − 62.77	388	59.42	55.54 − 63.21	141	66.82	60.03 − 73.14	89	62.68	54.17 − 70.64
Female	481	40.02	37.23 − 42.85	265	40.58	36.79 − 44.46	70	33.18	26.86 − 39.97	53	37.32	29.36 − 45.83
Age^a^												
0 − 19	156	13.47	11.56 − 15.57	18	2.92	1.74 − 4.58	1	0.48	0.01 − 2.63	69	50.36	41.7 − 59.01
20 − 39	168	14.51	12.53 − 16.67	71	11.53	9.11 − 14.32	23	10.95	7.07 − 15.98	36	26.27	19.13 − 34.48
40 − 59	355	30.66	28.01 − 33.4	219	35.55	31.77 − 39.48	82	39.05	32.41 − 46.0	21	15.33	9.75 − 22.47
60 +	479	41.36	38.51 − 44.26	308	50	45.98 − 54.02	104	49.52	42.57 − 56.49	11	8.03	4.08 − 13.91
Total	1 203	100.00		654	100.00		211	100.00		142	100.00	

95% *CI* confidence intervals of 95%

^a^Data not available in all cases (Neglected Tropical Diseases – Gender: 1, Age: 45; Schistosomiasis – Gender: 1, Age: 38; Chagas disease – Age: 1; Leishmaniases – Age: 5)

**Table 3:** Joinpoint regression analysis of mortality rates for Neglected Tropical Diseases by age group in the state of Sergipe, Brazil, 1980–2013

Age group	Rates per 100 000		*Joinpoints*	Trends			Entire period		Last 15 years	
	Start	Final		Period	APC	95% CI	AAPC	95% CI	AAPC^15^	95% CI
Crude mortality and age-specific rates										
0 − 19	0.31	0.25	0	1980 − 2013	3.31^a^	0.3; 6.4	3.31^a^	0.3; 6.4	3.31^a^	0.3; 6.4
20 − 39	1.77	0.66	0	1980 − 2013	−2.40^a^	−4.2; -0.6	−2.40^a^	−4.2; -0.6	−2.40^a^	−4.2; -0.6
40 − 59	10.60	3.11	2	1980 − 1993	2.84	−4.0; 10.1	−0.6	−12.5; 12.9	3.45	−2.2; 9.4
				1993 − 1996	−31.56	−83.1; 177.6				
				1996 − 2013	3.45	−2.2; 9.4				
60 +	12.93	11.63	3	1980 − 1989	−8.32^a^	−15.7; -0.2	0.4	−8.6; 10.3	6.46^a^	3.6; 9.4
				1989 − 1992	62.46	−33.8; 298.8				
				1992 − 1996	−32.90	−55.2; 0.5				
				1996 − 2013	6.46^a^	3.6; 9.4				
All (CR)	2.81	1.96	3	1980 − 1989	−8.80	−17.5; 0.8	0.1	−11.4; 13.1	5.64^a^	1.6; 9.8
				1989 − 1992	52.58	−56.2; 432.0				
				1992 − 1997	−22.61	−44.3; 7.6				
				1997 − 2013	5.64^a^	1.6; 9.8				
Schistosomiasis (CR)	2.37	0.68	3	1980 − 1989	−7.44^a^	−13.1; -1.4	−2.3	−9.9; 5.9	5.28^a^	1.0; 9.7
				1989 − 1992	47.21	−35.0; 233.2				
				1992 − 1998	−28.49^a^	−40.8; -13.7				
				1998 − 2013	5.28^a^	1.0; 9.7				
Chagas Disease (CR)	0.18	0.77	4	1980 − 1989	−12.70	−28.6; 6.7	4.7	−15.7; 30.0	9.7^a^	3.1; 16.7
				1989 − 1992	95.18	−77.8; 1613.6				
				1992 − 1995	−31.44	−77.7; 110.5				
				1995 − 2010	14.99^a^	10.2; 20.0				
				2010 − 2013	−7.68	−29.1; 20.2				
Leishmaniases (CR)	0.09	0.14	0	1980 − 2013	−0.11	−2.4; 2.2	−0.11	−2.4; 2.2	−0.11	−2.4; 2.2
Proportional mortality ratios										
0 − 19	0.08	0.20	0	1980 − 2013	7.04^a^	3.5; 10.7	7.04^a^	3.5; 10.7	7.04^a^	3.5; 10.7
20 − 39	0.73	0.30	0	1980 − 2013	−2.53^a^	−4.2; -0.8	−2.53^a^	−4.2; -0.8	−2.53^a^	−4.2; -0.8
40 − 59	1.43	0.56	2	1980 − 1993	2.95	−2.6; 8.8	0.0	−10.7; 12.0	5.11	−0.8; 11.4
				1993 − 1996	−33.30	−80.7; 130.8				
				1996 − 2013	5.11	−0.8; 11.4				
60 +	0.32	0.31	3	1980 − 1989	−7.34^a^	−13.9; -0.3	1.0	−7.5; 10.3	6.79^a^	3.7; 10.0
				1989 − 1992	58.97	−30.8; 265.3				
				1992 − 1996	−31.29	−53.8; 2.1				
				1996 − 2013	6.79^a^	3.7; 10.0				
All	0.43	0.35	3	1980 − 1989	−5.47	−13.3; 3.1	0.9	−9.5; 12.4	5.72^a^	1.6; 10.0
				1989 − 1992	42.32	−52.3; 324.8				
				1992 − 1997	−20.51	−41.9; 8.8				
				1997 − 2013	5.72^a^	1.6; 10.0				
Schistosomiasis	0.36	0.12	2	1980 − 1994	3.29	−0.3; 7.1	−0.9	−10.6; 9.7	4.98	−0.3; 10.6
				1994 − 1997	−40.21	−80.8; 86.4				
				1997 − 2013	4.98	−0.3; 10.6				
Chagas Disease	0.03	0.14	0	1980 − 2013	8.59^a^	6.8; 10.4	8.59^a^	6.8; 10.4	8.59^a^	6.8; 10.4
Leishmaniases	0.01	0.02	0	1980 − 2013	0.78	−1.5; 3.1	0.78	−1.5; 3.1	0.78	−1.5; 3.1

*APC* annual percent variation, *AAPC* average annual percent variation, *AAPC* ^*15*^ Annual percent variation in the last 15 years, *95% CI* confidence intervals of 95%, *CR* crude rate

^a^Statistically important

## Discussion

The results from the present study have shown that NTDs are a real and worrisome problem in the state of Sergipe. Until the early 90s, the mortality rates were decreasing, but, from this period on, they showed an increasing trend, mainly for the NTDs﻿ (joint analysis),﻿ Schistosomiasis and Chagas disease. Unlike a recently published study, which showed Chagas disease as the NTD responsible for the highest mortality in Brazil, followed by Schistosomiasis and Leishmaniases [12], especially in Sergipe, there in an inversion, and Schistosomiasis appears with the highest mortality, followed by Chagas disease and Leishmaniases.

In Latin America, Brazil and particularly the Northeast Region showed the greatest endemic areas for Schistosomiasis, despite the control measures, mainly based on periodical researches in feces of individuals in endemic areas, followed by treatment of the positive cases [12, 13]. These measures decreased the morbidity and mortality, but the disease was not eliminated, and continued to show significant mortality, such as the ones presented in our study [14, 15]. The factors that renders more difficulty in controlling the disease are population migration, unsafe sanitary conditions and appearance of new foci [12, 15]. This is not different in Sergipe, where variables such as temperature, low pluviometric indices, land use, land cover, and soil humidity are factors that contribute to the proliferation of Schistosomiasis and other neglected diseases. Schistosomiasis showed the highest mortality rates, similar to the State of Pernambuco, Northeast of Brazil [16], which predominates in most advanced ages, explained by the chronic nature of the disease and by its association with comorbidities, leading to more severe forms [14]. The lowest rates in younger age groups reflect the direction of the control and treatment programs for children and adolescents [17]. Despite the prevention and treatment programs, Schistosomiasis showed an increasing mortality trend in recent years. This is rather disturbing and reveals the need to prioritize surveillance measures related to the disease and investigation of the factors that are supporting the mortality, taking into account that Sergipe remains as an endemic area [18].

In Chagas disease, the number of deaths appeared higher in individuals over 40 years of age, which shows that infections probably acquired in the past turns prominent in in this age group [19], since acute infections do not play a significant role to mortality in Brazil. Due to the chronic nature of this entity and the increase in life expectancy of the population, it will probably remain high in the future [20]. There are important regional differences related to mortality that can be explained by the efficacy of control measures and vector eradication programs [21]. The latter is paramount in Sergipe, since disease vector has not been eradicated. The mortality trends in Sergipe are increasing and, similar to the mortality for Chagas disease in most areas of the Northeast and North of Brazil, it will remain as an important health problem in coming years [22, 23]. That will pose a great challenge for policy makers, to ensure greater coverage of control measures in the population. Thus, public health preventive actions are needed, followed by appropriate monitoring and evaluation. In spite of the efforts to reduce transmission, there is actually no impact in decreasing mortality rates [24, 25].

Visceral Leishmaniasis is the most common type in the Northeast Region and in Sergipe as well. There was a greater number of deaths in the age group 0–19, and subsequent reduction in the age group 60 plus, which may reflect a sub-notification in the number of deaths of elderly patients. Younger individuals are more prone to have their diagnosis established, rendering a great number of notifications in this age group [26]. The higher mortality rates in children may reflect greater contact with vectors and hosts, immature immunity, and nutritional deficiencies [27, 28]. Studies conclude that the high lethality produced by Leishmaniasis is due to, in addition to other factors, the lack of study on the efficacy of the drugs available in the market [29]. In Sergipe, the mortality trend is stable, despite the disease, vector and host control programs, which favors the need to prioritize measures of surveillance of disease transmission, and treatment.

Mortality rates for the NTDs were higher in elderly patients, who faces the chronic nature of the diseases and comorbidities, thus, contributing to worse outcomes. Besides, there has been some difficulties in the implementation public health policies in the state, and the mere existence of health units does not guarantee full and proper observation of the population demands [8].

The present study was conducted using secondary data, and it may show limitations by sub-notifications and diagnostic errors. Deaths due to NTDs' complications might not be registered correctly, especially if we consider that only the underlying cause of death was verified. Other causes of death, even citing NTDs in other lines were not included, which eventually underestimates the mortality rates. Other authors also faced limitations in research using secondary data [12, 20].

## Conclusions

In conclusion, NTDs continue to be important mortality causes in the state of Sergipe, showing increasing mortality trends in the last 15 years. It is necessary to revise public health policies for the control of these neglected diseases, especially to decrease those high mortality rates.

## Additional file

Additional file 1: Multilingual abstracts in the five official working languages of the United Nations. (PDF 555 kb)

## Abbreviations

ASRs: Age-standardized rates
CRs: Crude rates
HAT: Human African Trypanosomiasis
HDIs: Human Development Indices
IBGE: Instituto Brasileiro de Geografia e Estatística [Brazilian Institute of Geography and Statistics]
NTDs: Neglected tropical diseases
PMRs: Proportional mortality ratios
SIM: Sistema de Informações sobre Mortalidade [Mortality Information System]
WHO: World Health Organization
