# Supplementary material for: Mortality Trends for Neglected Tropical Diseases in the State of Sergipe, Brazil, 1980–2013
Source: Infect Dis Poverty. 2017 Feb 8;6:20. doi: 10.1186/s40249-016-0232-8 (PMC5297211; doi:10.1186/s40249-016-0232-8)
Supplement: Additional file 1: — Multilingual abstracts in the five official working languages of the United Nations. (PDF 555 kb) [file 40249_2016_232_MOESM1_ESM.pdf]

Translation of the abstract into the five official working languages of the United Nations

اتجاهات وفيات أمراض المناطق المدارية المهمَلة في ولاية سيرجيبي، البرازيل، 1980-2013

ماركوس أنطونيو كوستا دي البوكيرك، دانييل مينيزيس دياز، لوكاس تيكسيرا فييرا، كارلوس أنسيلمو ليما، أنجيلا ماريا دا سيلفا

الملخص

الخلفية: الأمراض الاستوائية المهمَلة هي مجموعة من الأمراض المعدية التي تؤثر على الوضع الاجتماعي والاقتصادي للسكان ، ولا سيما أن 1.4 مليار شخص يعيشون دون مستوى الفقر. وقد بحثت هذه الدراسة حجم اتجاهات الوفيات الزمنية لهذه الأمراض في ولاية سيرجيبي، بشمال شرق البرازيل.

الطرق: أجرينا دراسة بيئية من سلسلة زمنية، استناداً إلى البيانات الثانوية المستمدة من نظام معلومات الوفيات التابع لوزارة الصحة. تم حساب معدلات الوفيات (الصريحة ونسب العمر القياسية والمعدل النسبي) من الوفيات الناجمة عن أمراض المناطق المدارية المهمَلة في ولاية سيرجيبي، من عام 1980 إلى عام 2013. وقد تم الحصول على الاتجاهات الزمنية باستخدام نموذج الانحدار Joinpoint.

النتائج: تم اعتماد 306872 حالة وفاة في الولاية وتم ذكر أمراض المناطق المدارية المهمَلة باعتبارها السبب الأساسي في 203 1شهادة (0.39٪). وكان متوسط عدد الوفيات 35.38 في السنة، وكانت معدلات الوفيات الصريحة ومعدلات العمر القياسية، على التوالي: 2.16 لكل 100000 نسمة (95٪ فاصل ثقة: 1.45- 2.87) و2.87 لكل 000 100 نسمة (95٪ فاصل ثقة: 1.93- 3.82)، وكان معدل الوفيات النسبي 0.41٪ (95٪ فاصل ثقة: 0.27- 0.54). في تلك الفترة، تسبب مرض البلهارسيا في 654 حالة وفاة (54.36٪)، يليه داء شاجاس، بـ 211 (17.54٪)، وداء الليشمانيات، بـ 142 (11.80٪) حالة وفاة. إجمالي الأمراض الأخرى 196 حالة وفاة (16.30٪). كانت هناك زيادة معدلات وفيات لمرض البلهارسيا وداء شاجاس في السنوات الـ 15 الماضية، وفقاً لمعدلات العمر القياسية ، واستقرار لاتجاهات وفيات داء الليشمانيات.

الاستنتاجات: تُظْهِر أمراض المناطق المدارية المهمَلة اتجاهات متزايدة وهي مشكلة صحية حقيقية في ولاية سيرجيبي، وذلك لأنها مسؤولة عن معدلات وفيات كبيرة. وتستدعي الأمراض التالية الانتباه لإظهارها أكبر عدد من الوفيات في فترة الدراسة: البلهارسيا ومرض شاجاس وداء الليشمانيات. نقترح أخيراً أن يتخذ المديرون العموميون الإجراءات المناسبة لوضع استراتيجيات جديدة في المراقبة الوبائية والعلاجية، وفي متابعة هؤلاء المرضى.

Translated from English version into Arabic by Free bird, through

TRANSLATORS
WITHOUT BORDERS

1980-2013 年被忽视热带病在巴西塞尔希培州的死亡率发展趋势

Marcos Antônio Costa de Albuquerque, Danielle Menezes Dias, Lucas Teixeira Vieira, Carlos Anselmo Lima, Angela Maria da Silva

摘要:

引言：被忽视热带病是一类影响人类社会经济水平的传染性疾病，尤其是处于贫困线以下的 14 亿人口。本研究调查了巴西东北塞尔希培州地区被忽视热带病死亡率的变化趋势。

方法：根据来自巴西卫生部死亡信息系统的二级信息，进行生态学时间序列研究。根据塞尔希培州 1980-2013 年被忽视热带病死亡病例数计算死亡率(粗死亡率、标准化年龄死亡率、

构成比)。通过 Joinpoint 回归模型分析时间趋势。
结果:据统计，在塞尔希培州确认的 306 872 例死亡病例中,有 1 203(0.39%)例的死因为被忽视热带病。每年平均死亡病例数为 35.38，粗死亡率和标准化年龄死亡率分别为 2.16/10 万（95%*CI*：1.45-2.87），和 2.87/10 万（95%*CI*：1.93-3.82）；比例死亡比为 0.41%(95%*CI*：0.27-0.54)。在此期间，血吸虫病造成了 654 人死亡（54.36%)，紧接着是锥虫病，共计 211 例（17.54%)，利什曼病导致 142 例（11.80%)死亡，其他疾病共计 196 例（16.30%）死亡病例。标准化年龄死亡率的结果显示，在过去十五年里，血吸虫病和锥虫病死亡率增高，利什曼病的死亡率趋于稳定。
结论：被忽视的热带病发病呈上升趋势，同时因其高病死率已切实成为了塞尔希培州的一个公共卫生问题。下列疾病包括血吸虫病、锥虫病和利什曼病，因其在研究期间体现出的较高死亡率需引起广泛关注。最后，我们希望公共卫生管理者能在流行病学和治疗的监测，以及患者随访等方面采取适当行动从而制定新的防治策略。

Translated from English version into Chinese by Lu-Lu Huang, edited by Pin Yang

Tendances de la mortalité due aux maladies tropicales négligées dans l’état brésilien du Sergipe, 1980-2013

Marcos Antônio Costa de Albuquerque, Danielle Menezes Dias, Lucas Teixeira Vieira, Carlos Anselmo Lima, Angela Maria da Silva

Résumé
Contexte : Les maladies tropicales négligées sont un groupe de maladies transmissibles qui affectent les populations défavorisées du point de vue socioéconomique, et en particulier les 1,4 milliard de personnes qui vivent en dessous du seuil de pauvreté. Notre étude a examiné l’ampleur des tendances dans le temps de la mortalité liée à ces maladies dans l’état du Sergipe, dans le nord-est du Brésil.
Méthodes : Nous avons mené une étude écologique de séries temporelles, basée sur des données secondaires tirées du Système d’information sur la mortalité du Ministère de la Santé. Les taux de mortalité (bruts, ajustés par âge et ratio proportionnel) ont été calculés à partir des décès causés par les maladies tropicales négligées dans l’état de Sergipe entre 1980 et 2013. Nous avons obtenu les tendances dans le temps à l’aide du modèle de régression Joinpoint.
Résultats : Dans l’état du Sergipe, 306 872 décès ont été enregistrés sur la période et une maladie tropicale négligée a été indiquée comme cause du décès dans 1203 certificats (0,39 %). Le nombre moyen de décès était de 35,38 par an et les taux de mortalité bruts et standardisés par âge étaient, respectivement, de 2,16 pour 100 000 habitants (IC à 95 % : 1,45-2,87) et 2,87 pour 100 000 habitants (IC à 95 % : 1,93-3,82) ; le ratio proportionnel de mortalité était de 0,41 % (IC à 95 % : 0,27-0,54). Sur cette période, la schistosomiase a causé 654 décès (54,36 %), suivie de la maladie de Chagas avec 211 décès (17,54 %) et de la leishmaniose avec 142 décès (11,80 %). Les autres maladies ont causé au total 196 décès (16,30 %). La mortalité liée à la schistosomiase et à la maladie

de Chagas a eu tendance à augmenter au cours des 15 dernières années, selon les taux standardisés par âges, tandis que celle causée par la leishmaniose restait stable.
Conclusions : Les maladies tropicales négligées sont en progression et posent un réel problème de santé publique dans l'état du Sergipe car elles sont à l'origine d'une mortalité significative. Trois maladies retiennent en particulier l'attention, car ce sont elles qui ont causé le plus de décès pendant la période de l'étude : schistosomiase, maladie de Chagas et leishmaniose. Pour conclure, nous suggérons que les pouvoirs publics prennent les mesures nécessaires et développent de nouvelles stratégies de surveillance épidémiologique et thérapeutique et de suivi des patients.

Translated from English version into French by Suzanne Assenat, through

TRANSLATORS
WITHOUT BORDERS

Тенденции смертности для забытых тропических болезней в штате Сержипи, Бразилия, 1980-2013

Marcos Antônio Costa de Albuquerque, Danielle Menezes Dias, Lucas Teixeira Vieira, Carlos Anselmo Lima, Angela Maria da Silva

Реферат

Фон: Забытые тропические болезни, это ряд инфекционных заболеваний, которые влияют на потока населения социально-экономический статус, в частности, 1,4 млрд человек, которые живут ниже уровня бедности. Это исследование исследовало величину временных трендов смертности от этих болезней в штате Сержипи, Северо-Восточный Регион Бразилии.
Методы: Мы провели экологическое исследование временных рядов на основе вторичных данных, полученных из информационной системы смертности Министерства здравоохранения. Показатели смертности (сырой, возрасту частоты и пропорциональное соотношение) были рассчитаны от гибели из-за забытых тропических болезней в штате Сержипи, с 1980 по 2013 год. Тенденции времени были получены с помощью модели регрессии Joinpoint.
Результатаы: 306,872 умерших были освидетельствованы в государственной и Забытые тропические болезни были названы в качестве основной причины в 1,203 сертификатов (0.39%). Среднее число умерших составило 35.38 в год, а грубые и стандартизованные по возрасту коэффициенты смертности составили соответственно: 2.16 на 100 000 населения (95%*CI:* 1.45-2.87) и 2.87 на 100 000 населения (95%*CI*: 1.93-3.82); коэффициент пропорциональной смертности составлял 0.41% (95%*CI*: 0.27-0.54). В тот период, Шистосомоз причинил 654 смерти (54.36%), за которым следуют болезни Шагаса, с 211 (17.54%) и Лейшманиоз, 142 (11.80%) смертей. Другие заболевания в общем причинили 196 смертей (16.30%).
Заключение: Забытые тропические болезни показывают тенденцию к росту и являются реальной проблемой общественного здравоохранения в штате Сержипи, поскольку они

отвечают за значительную смертность. Следующие заболевания требуют внимания, показывая большое число смертей в период обучения: Шистосомоз, болезнь Шагаса и Лейшманиоза. Мы, наконец, предположим, что государственные менеджеры принимают надлежащие меры для разработки новых стратегий в эпидемиологической и терапевтической наблюдении, и в последующем у этих пациентов.

Translated from English version into Russian by Hao-Qi Zhang, through

Tendencias de mortalidad por enfermedades tropicales desatendidas en el estado de Sergipe (Brasil), 1980-2013

Marcos Antônio Costa de Albuquerque, Danielle Menezes Dias, Lucas Teixeira Vieira, Carlos Anselmo Lima, Angela Maria da Silva

Resumen
Contexto: Las enfermedades tropicales desatendidas son un grupo de enfermedades comunicables que afectan a la población de nivel socioeconómico bajo; en concreto, a 1.400.000.000 personas que viven por debajo del umbral de la pobreza. En este estudio hemos investigado la magnitud de la tendencia de mortalidad en el tiempo debida a dichas enfermedades en el estado de Sergipe, en el nordeste de Brasil.
Metodología: Llevamos a cabo un estudio ecológico de series temporales basado en datos secundarios derivados del Sistema de Información sobre Mortalidad del Ministerio de Sanidad. Las tasas de mortalidad (ratio aproximada, ajustada a la edad y proporcional) se calcularon en función de las muertes provocadas por enfermedades tropicales desatendidas en el estado de Sergipe, entre 1980 y 2013. Las tendencias en el tiempo se calcularon utilizando el modelo de regresión *joinpoint*.
Resultados: En el periodo del estudio, 306.872 muertes fueron certificadas en el estado de Segirpe. En 1.203 de ellas se mencionaron enfermedades tropicales desatendidas como causa subyacente (0,39 %), lo que se traduce en 35,38 muertes por año y una tasa de mortalidad aproximada de 2,16 por 100.000 habitantes (95 % *CI*: 1,45-2,87) y una tasa de mortalidad basada en la edad de 2,87 por 100.000 habitantes (95 % *CI*: 1,93-3,82); la tasa de mortalidad proporcional fue del 0,41 % (95 % *CI*: 0,27-0,54). En dicho periodo, 654 muertes se debieron a la esquistosomiasis (54,36 %), seguida de la enfermedad de Chagas, con 211 muertes (17,54 %) y la leishmaniosis, con 142 (11,80 %). El resto se debieron a otras enfermedades tropicales desatendidas (16,30 %). En los últimos quince años se revela una tendencia al alza en la mortalidad por esquistosomiasis y enfermedad Chagas, según las tasas basadas en la edad, mientras que la de la leishmaniosis se mantiene estable.
Conclusión: Las enfermedades tropicales desatendidas revelan tendencia al aumento y, puesto que son responsables de un gran número de muertes, constituyen un problema para la salud pública en el estado de Sergipe. La esquistosomiasis, la enfermedad de Chagas y la leishmaniosis se destacan por la cantidad de muertes causadas durante el periodo del estudio. Como colofón, recomendamos a la administración pública que tome las medidas oportunas para desarrollar nuevas estrategias de monitorización epidemiológica y terapéutica y de seguimiento de los pacientes afectados.

Translated from English version into Spanish by Marta Callava Linares, through

TRANSLATORS
WITHOUT BORDERS
